# Local Food Environments, Suburban Development, and BMI: A Mixed Methods Study

**DOI:** 10.3390/ijerph15071392

**Published:** 2018-07-02

**Authors:** Maureen Murphy, Hannah Badland, Helen Jordan, Mohammad Javad Koohsari, Billie Giles-Corti

**Affiliations:** 1 Centre for Health Equity, The University of Melbourne, Melbourne 3010, Australia; 2 Centre for Urban Research, RMIT University, Melbourne 3000, Australia; hannah.badland@rmit.edu.au (H.B.); billie.giles-corti@rmit.edu.au (B.G.-C.); 3 Centre for Health Policy, The University of Melbourne, Melbourne 3010, Australia; h.jordan@unimelb.edu.au; 4 Faculty of Sport Sciences, Waseda University, Saitama 359-1192, Japan; javad.koohsari@baker.edu.au; 5 Behavioural Epidemiology Laboratory, Baker Heart and Diabetes Institute, Melbourne 3004, Australia; 6 Mary MacKillop Institute for Health Research, Australian Catholic University, Melbourne 3000, Australia

**Keywords:** food environment, urban planning policy, obesity, mixed methods, cities, urban health

## Abstract

More than half the world’s population now live in urban settlements. Worldwide, cities are expanding at their fringe to accommodate population growth. Low-density residential development, urban sprawl, and car dependency are common, contributing to physical inactivity and obesity. However, urban design and planning can modify urban form and enhance health by improving access to healthy food, public transport, and services. This study used a sequential mixed methods approach to investigate associations between food outlet access and body mass index (BMI) across urban-growth and established areas of Melbourne, Australia, and identify factors that influence local food environments. Population survey data for 3141 adults were analyzed to examine associations, and 27 interviews with government, non-government, and private sector stakeholders were conducted to contextualize results. Fast food density was positively associated with BMI in established areas and negatively associated in urban-growth areas. Interrelated challenges of car dependency, poor public transport, and low-density development hampered healthy food access. This study showed how patterns of suburban development influence local food environments and health outcomes in an urbanized city context and provides insights for other rapidly growing cities. More nuanced understandings of the differential effect of food environments within cities have potential to guide intra-city planning for improving health and reducing inequities.

## 1. Introduction

More than one half the world’s population live in urban settlements [1] and this number is set to almost double by 2050 [2]. Worldwide, cities are expanding at their urban fringe to accommodate population growth, while also becoming more densely populated within [1]. The United Nations’ New Urban Agenda recognizes that population growth concentrated in cities is placing significant pressure on housing, infrastructure, food systems, the natural environment, and services [2]. However, the World Health Organization suggests that these challenges also bring opportunities to achieve good health through sustainable urban development [1]. 

Urban design and planning policies can enhance population health by: improving access to healthy affordable food; prioritizing public transport, walking, and cycling as travel modes; and supporting compact higher-density residential development [1,3]. While there is potential to improve population health through such policies, low-density residential development, urban sprawl, and car dependency are common and have contributed to increases in physical inactivity, overweightness, and obesity [4,5]. For example, a review found that urban sprawl and less land use mix in North America were positively associated with increased body weight [4]; and in Australia, more sprawling suburbs were positively associated with insufficient physical activity, overweightness, and obesity [6]. Furthermore, a UK study of 420,000 adults found that high residential density was associated with lower body mass index (BMI), waist circumference, and body fat [7]. Therefore, increasing residential density and minimizing urban sprawl may have a protective effect on overweightness and obesity [5,7].

Many studies have investigated how the location of food outlets influences dietary intake and diet-related health outcomes, hypothesizing that a high level of access to supermarkets is protective of health and that a high level of access to fast food outlets is detrimental to health [8,9]. Indeed, evidence exists that supermarket access is negatively associated with body weight [9,10] and that fast food outlet access is positively associated with body weight [11,12]. Researchers have also investigated whether access to food outlets impacts dietary and health outcomes differently across urban, suburban, and rural environments [13,14]. For example, a U.S. study found varying associations between food environments and obesity depending on metro or nonmetro location, with fast food access positively associated with obesity in metro areas, but inversely associated in nonmetro areas [15]. A study from Denmark found that fast food proximity was positively associated with fast food intake; however, the magnitude of the association varied by urban, suburban, or rural location [13]. 

In Australia, many studies have investigated associations between food outlet access and dietary or health outcomes in urban [16] and regional areas [17]; however, little is known about food environments across established residential and urban-growth areas within cities. In the context of rapid urban growth, it is important to understand how patterns of suburban development influence local food environments and health outcomes. In the decade up to June 2016, more than three-quarters of Australia’s population growth was concentrated in capital cities, with Melbourne in the state of Victoria accounting for the largest growth [18]. Within Melbourne, local government areas on the fringe experienced some of the largest and fastest growth, and this trend is projected to continue [18]. At the same time, the delivery of infrastructure and services in new residential developments lags behind population settlement by many years [19]. Uneven suburban development across established and urban-growth areas of Melbourne may influence food environments and health outcomes; however, this is an understudied research area in an Australian context. A more nuanced understanding at the intra-city level has potential to guide and target urban design and planning policy interventions to specific geographic areas where health and access inequities exist [20]. 

Therefore, a mixed methods approach was used to investigate this relatively unknown research area. Mixed methods can be used to bring together a more comprehensive account of a research area and can improve the usefulness of findings for practitioners [21]. While focused on an Australian city, this research has relevance for other cities experiencing rapid population growth and suburban development and contributes to the food environment evidence base utilizing mixed methods approaches. Accordingly, the aims of this study were to: (i) investigate associations between measures of supermarket and fast food chain access and BMI across established and urban-growth areas; (ii) understand the contextual factors that influence food environments in established and urban-growth areas; and (iii) identify challenges to the development of healthy equitable local food environments.

## 2. Materials and Methods 

A sequential mixed methods research design was applied to investigate the research aims [22]. First, quantitative and spatial analytical methods were used to investigate access to food outlets across established and urban-growth areas and associations with BMI. Second, key informant interviews were conducted to contextualize, explain, and triangulate findings [21,22]. Mixing of the approaches occurred during data collection and at interpretation [22], whereby the quantitative and spatial analyses guided the sampling strategy for the qualitative strand, and the qualitative strand assisted in interpreting and contextualizing the quantitative results.

### 2.1. Quantitative Strand

**Figure 1:**
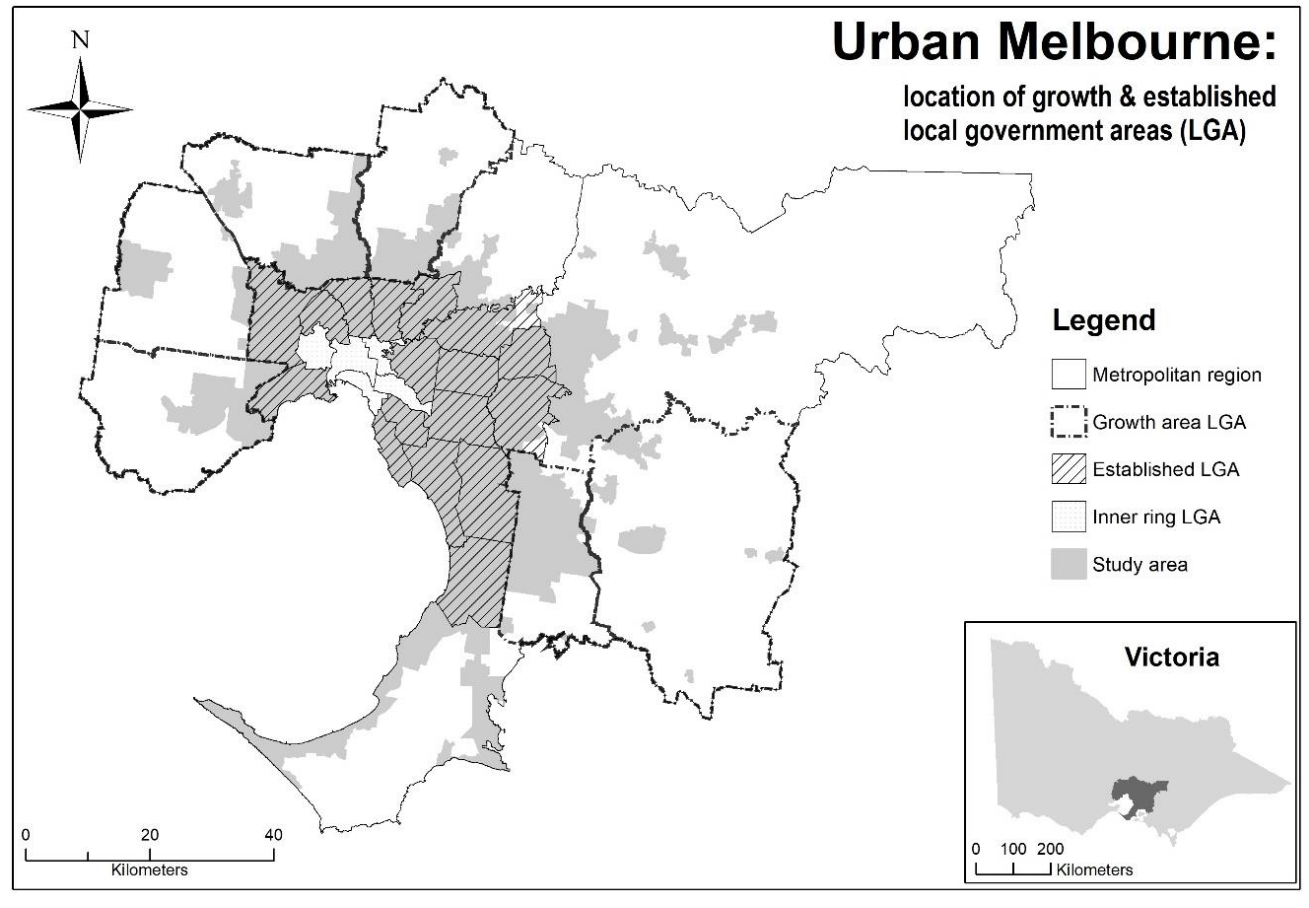
Study area.

Data were obtained from the preventive health survey (PHS) 2012/13, a self-report population health dataset collected by the Victorian Department of Health and Human Services. Full details of the survey have been reported elsewhere [23]. Briefly, the aim of the PHS was to assess the prevalence of health risks among adults who were cluster-sampled from 23 municipalities across Victoria. Of the 9806 adult respondents, 6707 (68.4%) were successfully geocoded at the residential address level. In the present study, the sample drawn from the PHS comprised 3141 respondents from the Melbourne metropolitan region. With a focus on urban food environments, the study was delimited to urban areas of greater Melbourne. Two categories of suburban development were created: established area and urban-growth area municipalities. There were 1648 respondents residing in six established area municipalities in the middle-outer ring of Melbourne, and 1493 residing in six urban-growth area municipalities on the fringe (Figure 1). It is estimated that 42% of population growth from 2011–2031 will occur in the six urban-growth area municipalities of greater Melbourne and a seventh growth area outside the metropolitan region [19]. The PHS received ethics approval from the Victorian Department of Health and Human Services (02/12) and University of Melbourne (1441599.1).

#### 2.1.1. Outcome Variable

The outcome variable was BMI (kg/m^2^), calculated using self-reported height (m) and weight (kg). Consistent with previous studies [24], 16 pregnant respondents and 12 respondents with extreme BMI values (≥50 kg/m^2^) were excluded from the analysis. Additionally, 239 respondents with missing height and/or weight data were removed prior to analysis.

#### 2.1.2. Covariates

Individual-level covariates comprised age, gender, education, and household income. Behavioural covariates comprised self-reported vegetable intake (serves/day), fruit intake (serves/day), fast food (such as burgers, pizza, hot chips) intake (frequency/fortnight), soft drink intake (frequency/fortnight), smoking status (current smoker/not a smoker), and physical activity. A physical activity variable for total activity per week was created by summing minutes per week of walking and moderate and vigorous physical activity (doubled prior to summing), then categorizing into: no activity (0 min); <150 min per week; and ≥150 min per week. All data were truncated at 180 m per day [25]. In accordance with guidelines recommending that physical activity is accrued on most days of the week [26], a categorical variable was created for sedentary (0 min/week); insufficient time and/or sessions (<150 min or ≥150 min and <5 sessions); and sufficient time and sessions (≥150 min and ≥5 sessions). Respondents with missing physical activity (*n* = 113) and/or age and education (*n =* 36) data were excluded from the analysis.

The Index of Relative Socio-Economic Disadvantage (IRSD) was included as an area-level covariate. The Index of Relative Socio-Economic Disadvantage aggregates the socio-economic census data of individuals within a geographic area, including variables such as low income, educational attainment, unemployment, disability, single-parent households, and low English-language proficiency [27]. Thirteen respondents lived in areas without an IRSD score and were excluded from the analysis.

#### 2.1.3. Exposure Variables

The exposure variables were supermarket access and fast food chain access. Consistent with previous studies, access to supermarkets was used as a proxy for ”healthy food” access in the analysis. Supermarkets provide the range of foods required for a healthy diet [28,29], including affordable fruit and vegetables [30], and access to a supermarket has been shown to be associated with diet quality [31] and healthier weight [9]. In Australia, the top four major supermarket chains (i.e., Woolworths, Coles, IGA, and Aldi) account for almost 90% of the market share [32]. A commercial supermarket dataset was purchased [33] and extensively cross-checked and verified against company websites, the White/Yellow pages, and Google Street View. The top six fast food chains in Australia (i.e., McDonald’s, Kentucky Fried Chicken, Subway, Hungry Jack’s, Domino’s Pizza, and Red Rooster) were used as a proxy for “unhealthy food” access. Fast foods are energy-dense and nutrient-poor, and eating at least one fast food meal a week is associated with weight gain, overweightness, and obesity [34]. Together, these fast food restaurants (not including Domino’s Pizza) account for more than 75% of chained fast food value share [35]. Fast food chain data were sourced from an open access directory of geocoded businesses (www.zenbu.org) [36], company websites, and the White/Yellow pages and extensively cross-checked and verified. There were 742 supermarkets and 648 fast food chain outlets in the datasets within 10 km of the metropolitan Melbourne boundary.

Food outlet density and proximity measures were created using geographic information systems software (ESRI, Redlands, CA, USA). The origin-destination (OD) Matrix tool in the Network Analyst extension was used to calculate the density of outlets around each participant’s geocoded address using network-based buffers ranging from 800 m to 3000 m. Road network data were sourced from VicMap Transport [37]. A “pedestrian road network” was created to model a walkable distance of between 10 and 20 min by including walking and bike paths and removing tollways/freeways. Fast food outlets at tollway/freeway service centres and at Melbourne airport were excluded because they were not pedestrian-accessible. The pedestrian road network was used in analyses at 800 m, 1000 m, and 1600 m buffers, and the unmodified “car road network” used at the 2000 m and 3000 m buffers. The selection of buffers has been described in full elsewhere [23]. In brief, the density of supermarkets has been found to be associated with lower BMI within 800 m, 1000 m, and 2000 m buffers [23,38]. The density of fast food outlets has shown significant associations with higher odds of obesity within 1000 m and higher BMI within 1600 m buffers [39,40]. In addition, travel-mode survey data for 2013 in Melbourne showed the median distance travelled from home to a supermarket was 0.7 km by walking, 2.7 km by car, and 2.2 km by all modes including public transport; and the median distance travelled to a fast food outlet was 0.5 km by walking, 3.9 km by car, and 3.3 km by all modes [41]. Proximity measures for supermarket access and fast food chain access were created along both road networks. Closer proximity to supermarkets has shown significant associations with decreased obesity prevalence [42], and increased distance to fast food outlets has shown significant associations with decreased BMI in women [43].

#### 2.1.4. Statistical Analysis

Population-weighted means and standard errors for individual-level and behavioural covariates and outcome variables were calculated using the population size and the number sampled in each sampled municipality. Descriptive statistics for local food environment access measures were calculated using means and standard deviations for density measures and the median and interquartile range for proximity measures. Data were stratified by established versus urban-growth municipality location. Tests of statistical significance across strata were calculated to compare means and proportions. Generalized estimating equations were fitted to model associations between BMI and food outlet density and proximity, adjusting for clustering at the municipality level. All analysis was conducted in Stata version 13.0 (StataCorp, College Station, TX, USA), and the level of statistical significance was α < 0.05.

### 2.2. Qualitative Strand 

#### 2.2.1. Sampling and Recruitment

Qualitative interviews were conducted from December 2016 to August 2017. Purposive criterion sampling was used to select three middle-outer established area municipalities and two urban-growth area municipalities as information-rich cases for in-depth study [22]. The quantitative and spatial results guided the selection of municipalities for the qualitative strand, focusing on those that had a high proportion of dwellings with poor supermarket access (more than 1 km) in areas of high disadvantage (data not shown). Previous research has shown that supermarket access within 1 km was protective of BMI for people residing in high-disadvantage areas of Melbourne [23]. Purposive sampling of extreme cases can provide insights that can be used to guide remedial policy and other interventions [22].

Local government participants at the executive or managerial level were identified from Council websites, a social networking website (www.linkedin.com), and the authors’ networks. Urban planning, economic development, and public health portfolios were targeted for participant recruitment because these stakeholders have potential to influence local food environments. State government, non-government, and private sector participants were identified through websites and the authors’ networks. Snowball sampling was also used, whereby participants were asked to identify other people who would be meaningful to interview [44]. Participant recruitment occurred via email with a plain language statement and consent form attached. Ethics approval for the qualitative strand was granted by the University of Melbourne (1648132.1).

#### 2.2.2. Key Stakeholder Interviews

Semi structured interviews were conducted to gain stakeholder perspectives on the factors that influence the development of local food environments across established and urban-growth areas. All interviews, except one, were conducted face-to-face by the first author (Maureen Murphy). Participants were provided with a two-page report on the quantitative findings and maps that visualized the spatial distribution of supermarkets and transport access across metropolitan Melbourne. The interview was guided by thematic questions to explore roles, responsibilities, and factors that influence decision-making on food outlet access [45]. The interview guide is available in Table S1.

#### 2.2.3. Data Analysis

Participants were coded according to the level of government, sector, and portfolio. Transcriptions and initial data analysis were undertaken during the interview period by the first author (Maureen Murphy). The initial analysis was discussed by three coauthors (Helen Jordan, Hannah Badland and Billie Giles-Corti). Open coding and focused coding were used to assign data into categories [45,46]. In the present study, the data were interrogated using thematic coding and content analysis relating to established and urban-growth areas [46]. The coding framework and themes were revised and agreed by four co-authors (Maureen Murphy, Helen Jordan, Hannah Badland and Billie Giles-Corti). Excerpts from the interviews are presented to illustrate the contextual factors and challenges identified by the denoted state government (SG), local government (LG), non-government (NG), and private sector (PS) participants. Data analysis was undertaken using NVivo 11 (QSR International, Melbourne, Australia). 

## 3. Results

### 3.1. Quantitative Strand

#### 3.1.1. Descriptive Analysis

Descriptive statistics of PHS survey respondents are presented in Table 1. Respondents living in urban-growth areas were younger (*p* < 0.001) and less well-educated (*p* < 0.001) than respondents in established areas; however, more were employed (*p* < 0.001), and their household incomes were higher (*p* < 0.001). Mean BMI for urban-growth area respondents was higher (*p* = 0.030) and they had lower daily fruit consumption than respondents in established areas (*p* = 0.032). Fewer growth area respondents resided in either high- or low-disadvantage areas (*p* < 0.001), with 40% residing in mid-disadvantage areas, compared with 28% of established area respondents.

Compared with urban-growth area respondents, those residing in established areas had significantly greater access to supermarkets for all density measures, except within the 1000 m buffer, and were in closer proximity to supermarkets. Those residing in established areas had significantly greater access to fast food chains for all density measures and were in closer proximity than those residing in urban-growth areas (Table 2). 

#### 3.1.2. Associations with BMI

Supermarket density and proximity were not associated with BMI for any access measures across established and urban-growth area location (Table S2). Fast food chain density within 800 m (*p* = 0.047) and 1000 m (*p* < 0.001) buffers was associated with higher BMI for respondents in established areas. For each additional fast food chain, mean BMI increased by 0.20 kg/m^2^ (95% CI, confidence interval (0.00, 0.40)) and 0.27 kg/m^2^ (95% CI (0.14, 0.40;), respectively. In contrast, fast food chain density was associated with lower BMI for respondents in growth area municipalities within 800 m (*p* = 0.043) and 1600 m (*p* = 0.002) buffers (Table 3). Significant associations were found at 1600 m in unstratified analysis such that mean BMI decreased by 0.15 kg/m^2^ (95% CI (−0.27, −0.02)) for each additional fast food chain outlet. Significant associations were also found within the 3000 m buffer for all respondents; however, the effect size was too small (−0.08 kg/m^2^) to be of practical significance. Fast food chain proximity was not significantly associated with BMI in either established or urban-growth areas.

### 3.2. Qualitative Strand

Twenty-seven participants from state, local, and non-government and private sector organisations were interviewed. More than half of the participants (*n* = 15) had expertise in urban planning, followed by participants with public health and community planning (*n* = 8), urban economics/economic development (*n* = 2), and coordination/partnership development (*n* = 2) expertise. The average duration of interviews was 50 min.

Data from the qualitative interviews provided insights that assisted in understanding and interpreting the results of the quantitative and spatial analyses, specifically: (i) contextual factors that influence food outlet access in established and urban-growth areas; and (ii) urban planning and design challenges to the development of healthy local food environments.

#### 3.2.1. The Context

Participants provided contextual information about the development of food environments in urban Melbourne and how this differed across geographic areas. Melbourne was described as a city experiencing rapid population growth “higher than [in] London and matching New York in absolute numbers” (PS2), and that this population growth was accommodated by both urban expansion and densification. Participants reported population growth expanding outward in low-density residential developments in urban-growth areas, alongside increased residential density with “infill development” (SG4) in established areas. Both patterns of suburban development were regarded as key factors that influenced local food environments. 

In low-density residential areas, a state government participant described the “old model” of retail development that involved “placing your big shopping centre here…and some piddly little centres five or eight kilometres away that service the local catchment but never really thrive…so people just keep driving to the big sub-regional centre all the time” (SG8). Under this model, large retail centres were surrounded by extensive car parking where “the old-school line of thought is always cars front and centre, 100% about the car” (PS1). 

In contrast, in residential areas where infill development was occurring, the local food environment developed differently. When presented with spatial data from the quantitative strand, an established area stakeholder noted how increasing residential density can lead to improvements in food access:

*Twenty years ago, it* [middle-ring suburb] *was struggling, it had a fairly underperforming main street, it probably didn't feel safe, it certainly didn't look vibrant. The only thing that's changed in that street or around it is that there has been a lot more one and two-bedroom apartments built that's brought a new population in… [increasing density] brings opportunities: there's now two or three thousand additional people that have much better access to public transport, much better access to food, restaurants and places to meet. LG13*

However, increasing residential density was described as “a really hard sell” despite evidence that “if you move from 17.5 households per hectare up to 21 or 25, you get this burst of services appearing within your local fabric” (SG7). Another state government participant concurred:

*You've got to have a decent density—15 or 18 dwellings per hectare I don't think cuts it— you've got to have 20 to 25* [dwellings per hectare]… *in the vast majority of your conventional, residential areas. Then you've got to build it up as you get closer to your activity centres. SG8*

In summary, rapid population growth has been accommodated by low-density residential development and expansion in the urban-growth areas and increasing residential densities in established areas. These patterns of suburban development provided the context for the development of local food environments within Melbourne. Urban planning and design policies were identified as critical elements that can influence and modify food outlet access and potentially improve diet-related health outcomes. The following section explores the challenges to the development of healthy local food environments.

#### 3.2.2. The Challenges

##### Urban-Growth Areas

Many participants identified car-dependent retailing as an issue in urban-growth areas. Car dependency was linked to the lack of public transport in new residential developments, described as the “latent vulnerability of Melbourne”, where people with “high mortgage vulnerability and high car dependency [live] on the periphery, a long way from the jobs and no public transport options” (PS2). Participants referred to data presented during stakeholder interviews that reported on a Victorian urban planning guideline for growth areas that recommended that 80–90% of dwellings should be within one km of an activity centre anchored by a supermarket [47]. The data showed that only 26% of growth area dwellings were within 1 km of a supermarket, far less than the policy guideline [23]. 

State government participants agreed that car dependency and the lack of active and public transport infrastructure were obstacles to healthy food access in urban-growth areas; however, there was also the view that “it takes a while for a place to evolve” (SG4) and that eventually these places would have “the best access to supermarket retailing, both in terms of provision and in terms of transport access, walkability, cycling, road access, and connectivity” (SG2). A private sector participant pointed to the important role of the urban planning guideline on fresh food availability because “these PSPs [precinct structure plans] are nominating where the supermarkets can go” (PS1). Despite the master-planned environment of urban-growth areas, local government participants described the challenges they faced in implementation:

If you try and do a structure plan for an area and say we should have a corner shop here, we should have a moderate sized activity centre there, understanding all of the research around walking, cycling, and how people use their retail. When the time comes for the owner of what is usually a large greenfield parcel to find a buyer for that land, the perception is either the housing's not here yet, or that the population's not sufficient to support that… therefore they come back to council and they want to rezone it for housing. LG9

Stakeholders noted new “main street” (LG11) pedestrian-oriented retail developments in urban-growth areas that had active street frontages supporting walking and cycling, with car parking placed behind the main street. However, this type of urban design had met resistance from a major retailer who opposed efforts “to change [their] building footprint and the sea of car parking around it” (LG9).

The early delivery of services and infrastructure in new residential developments was seen as critical for health and wellbeing, because “habits of people are formed, especially in these new growth areas, in the first number of years.” (PS1). The timely provision of services was a significant challenge, as described by a local government participant:

Whilst we can reserve land for an activity centre or a shopping centre, we have no powers to ensure that a supermarket is developed there in a timely manner. We have a statutory planning team who will work with developers, and often with a supermarket they're going to need a population base… unfortunately in some areas of our municipality, development is slow and there can be quite a long lag between a development commencing and a supermarket being provided. LG5

##### Established Areas

Local and state government participants noted that middle-outer suburbs also experienced interrelated challenges of car dependency, poor public transport, and a poor urban design legacy that hampered healthy food access:

*Many of the older development areas, many of them probably 1950s to 1970s subdivisions,* [tend] *to have a lot of court bowls* [cul-de-sacs]*, which reduce penetration of public transport and increase walking distance to supermarkets. LG10*

Participants described the postwar period when “we were essentially designing residential areas around the motor car” (LG13). However, stakeholders reported it was “more of a challenge to retrofit effectively” (LG7) in the middle-outer suburbs. Therefore, efforts to improve healthy food access were constrained by the lack of “large land holdings that can be configured as supermarkets” (SG7). This led to geographic areas of poor access that were difficult to redress, as described by this local government participant:

There are gaps and there always will be some gaps especially in our established areas where it's not possible to retrofit our existing urban areas with new supermarkets. LG5

Further, state and local government participants reported significant socio-economic disadvantage in middle-outer suburbs that coincided with geographic areas of poor public and private transport access. Hence, poor access to healthy food outlets and car-dependent urban design was compounded by socio-economic disadvantage. 

##### Fast Food Access Across Established and Urban-Growth Areas

Efforts to improve food environments by restricting fast food outlets were discussed, with local government and state government participants noting that fast food outlets had been permitted in residential zones for more than 20 years, when located on a main road.

*It was known as a McDonald's amendment. It was a big case that* [established area council] *fought to try and stop the Macca's …* [however, the Planning Minister] *put through a number of changes into the planning system, where if you were of a certain configuration and met these requirements in the planning scheme, you still need a permit but you met all these ‘’as of right” requirements. So, it was a lot harder for councils and objectors to argue against it. SG8*

Several participants referred to a planning decision in outer Melbourne, where a fast food chain won an appeal to obtain a planning permit, despite strong opposition from the council and the community [48]. Against this background, one participant argued there should be a mechanism in the planning law to enable local government “to limit the proliferation of chain fast food outlets in communities” (NG3). A central issue raised by local government and non-government participants was that the planning legislation “doesn’t say that we must consider the health of Victorians” (NG2). Hence, local government had limited capacity to restrict the availability of unhealthy food outlets:

*If somebody was to apply for an application for a fast food place and we were to refuse it on the grounds that it's unhealthy, in basic terms, our likelihood of being able to get support through VCAT* [the planning tribunal] *is probably going to be very low. LG7*

Furthermore, in the planning tribunal environment, one participant noted the considerable burden of self-representation:

*Part of the issue is around the onus of proof being on the community and others to show harm from the placement of these* [fast food] *outlets, whereas it would be much fairer if these large entities who are creating demand show that they're not doing harm. NG3*

Restricting fast food access in residential areas in both established and growth areas was identified as a significant challenge, and communities were hampered by the urban planning legislative framework. 

## 4. Discussion

Over recent decades, rapid population growth in Australian cities has been accommodated by both low-density development on the urban fringe and increased residential densities in established areas [49]. While previous Australian studies have investigated the differential effects of food environments across socio-economic areas [23,50], little is known about how variations in residential density and suburban development within Australian cities influence food environments and health. 

This study used quantitative and qualitative approaches to investigate local food environments across established and urban-growth areas in Melbourne, Australia. Access to supermarkets and fast food chains was found to be significantly greater in established areas across most density and proximity measures. Furthermore, the median distance to the nearest supermarket was less than the median distance to the nearest fast food chain across all areas. Several U.S. studies have investigated food outlet access across urban, suburban, and rural areas [51,52], and a New Zealand study found that travel time to fresh food outlets within the city of Christchurch varied from less than one minute in the city centre to 20 min on the urban–rural fringe [53]. While variations in spatial access to food outlets between urban and rural areas is expected [51,53], it is the intra-city variation that may lead to differential health outcomes within cities, and has implications for local urban planning and design policy. To our knowledge, no Australian research has investigated food environments and BMI across areas of varying suburban development within cities. The present study found that respondents in the urban-growth areas had higher BMI than those living in established areas. These results are similar to those of a South Australian study that found that living further from the city centre was associated with increased waist circumference, suggesting that a high level of car commuting may be a contributing factor [54].

While no associations between residential access to supermarkets and BMI were found in this study, associations were detected between BMI and fast food density in both directions. For established area respondents, BMI increased as the number of fast food outlets increased within the 800 m and 1000 m buffer, in keeping with the hypothesis that fast food access is detrimental to BMI. Unexpectedly, fast food outlet density had a protective effect on BMI for respondents in growth areas within 800 m and 1600 m buffers. A U.S. study reported a similar outcome, where higher density of fast food outlets in nonmetro areas was associated with lower obesity rates [15]. They hypothesized that fast food outlets in nonmetro areas were located along major highways and interstate routes and were therefore utilized by travelers and visitors to the area, rather than residents. It is possible that the fast food chain outlets in the present study did not reflect a residential neighbourhood food environment for growth area respondents either. A longitudinal study of residents in a new development in an urban-growth area of Melbourne found that over one-third of residents spent two to three hours commuting to work each day [55]. With considerable time spent commuting and at work, the residential food environment may not be where food is purchased. A second possibility is that residents in urban-growth areas have lived in the area for a shorter period of time than residents of established areas, and therefore their weight status is influenced by a prior residential environment [56]. As the PHS dataset did not include a variable indicating length of residency, the analysis did not adjust for this possibility.

Stakeholder interview data pointed to poor public transport access and high car dependency as a major challenge to healthy food access in urban-growth areas. A study in a new urban-growth area development in Melbourne found that it was more than 30 months before the first residents had access to public transport to the shopping centre located five kilometres away [55]. Other studies have noted the considerable delay in policy implementation in new residential developments and have called for the early provision of infrastructure and services to support health outcomes [57,58,59]. For example, a longitudinal study in the Western Australian capital city Perth found that three years after relocating to a new development, more than two-thirds of residents still did not have access to destinations for daily living, including supermarkets, within 1600 m of household location [58].

Limiting fast food chain access was a common challenge experienced by stakeholders in both established and urban-growth areas. The absence of public health objectives in the urban planning legislation was identified as a barrier when objecting to fast food planning applications. Furthermore, stakeholders noted that the onus of proof was on communities to demonstrate that the fast food outlet would be detrimental to health. The literature suggests that land use regulation can be used to restrict fast food access [60,61,62]; however, evidence is still emerging. An evaluation of a Los Angeles zoning regulation found little change in the food environment or effects on dietary intake or obesity; however, the authors suggested this may have been because the study timeframe was too short [63]. Furthermore, they noted that the zoning regulation applied to standalone fast food outlets, which tend to be located on major roads and cater for customers who are driving through [63].

Residential density was identified as a key factor that influenced the food environment. The metropolitan planning strategy *Plan Melbourne 2017–2050* identified a residential density target of more than 20 dwellings per hectare to “help create stronger, healthier communities” [64] (p. 51); however, the average dwelling density across Melbourne has been assessed as 14 dwellings per hectare [65]. Our qualitative data suggested that building higher residential densities may be a challenge with “a lot of fear and concern about [urban] growth and development” (LG13), a finding echoed in other Australian cities [66]. The importance of higher residential densities for BMI was demonstrated in a U.K. study where 18 dwellings per hectare (1800 dwellings per km^2^) was identified as a threshold for stimulating better weight-related health outcomes [7]. While acknowledging that urban planning policies promoting densification may contribute to improved weight outcomes, it is important to note potential drawbacks, including air and noise pollution, urban heat island effects [3,67,68,69], increases in road trauma [70], overcrowding, and crime [71].

Compounding the problem of low-density residential development and car-dependent urban design, the present study found that middle-outer established areas of Melbourne that were once outer suburbs were experiencing concentrated disadvantage. In the present study, a higher proportion of respondents in established areas had annual household incomes less than $50,000 and were living in high-disadvantage areas compared with urban-growth area respondents. This was triangulated with qualitative data that pointed to greater socio-economic disadvantage in older middle-outer established areas. Previous research has shown that two middle-outer ring municipalities of Melbourne experienced the highest levels of poor supermarket access and area-level disadvantage when compared with all municipalities [23]. Several studies have documented this shift in the socio-spatial distribution of disadvantage in Australian cities over recent decades, from inner-urban to middle and outer-ring suburbs built in the postwar period [72,73]. Therefore, we offer three recommendations based on the present study.

First, there is a need for a review of the Victorian urban planning legislative framework to include public health considerations when assessing applications, and to remove the anomaly that allows fast food outlet provision in residential areas. Amending the legislation will assist local government and communities that seek to improve healthy food outlet access or restrict access to fast food outlets. Furthermore, the onus of proof should be reversed so that planning applicants for fast food fast outlets are required to show that a new outlet will not cause harm to residents. As an example, the Western Australian government introduced a provision to the *Liquor Control Act 1988* whereby liquor licence applicants need to show that the application is in the public interest and would not cause harm or ill-health to people, including “at-risk” groups [74]. Second, the Victorian urban planning policy supermarket access guideline should be implemented across all urban Melbourne, both established and urban-growth areas, with priority in disadvantaged areas. Currently, the policy guideline is only implemented in the six urban-growth municipalities of the 31 municipalities of Melbourne. Targeted urban planning policy interventions in geographic areas within cities is warranted to ameliorate inequities [20,75]. A UK study suggested that improved supermarket access combined with interventions to address socio-economic disadvantage may be effective in reducing obesity [76]. Third, there is a disjuncture between urban planning policy that provides guidance for supermarkets in new growth areas based on spatial catchments and the actual establishment of new supermarkets by the private sector based on population catchments. Urban planning and health policy interventions to improve healthy food access should be considered in new residential developments when supermarket development lags. These could include the provision of public transport to the nearest supermarket [55], “green cart” vendors [62], mobile produce markets [77], and tax incentives for supermarkets [78].

This study has limitations. First was the use of cross-sectional data, and therefore the associations detected may be biased by resident self-selection into areas that match food preferences, which might explain the observed differences in BMI. A recent U.S. longitudinal study found no evidence that geographic access to supermarkets or fast food outlets was associated with changes in BMI and suggested that self-selection may be biasing associations found in cross-sectional studies [79]. Second, the associations may be biased by using self-reported height and weight data. However, Australian studies found that self-reported height and weight data to calculate BMI is appropriate for middle-aged and older Australian adults [80]. Third, the food outlet dataset did not include all food outlets, potentially underestimating the food environment where people purchase food. The in-store food environment was also not considered, which may have contributed to observed differences in BMI. An Australian study found that urban-fringe and nonmetropolitan supermarkets had less shelf space for fruit and vegetables and more shelf space for soft drinks compared with urban supermarkets [81]. Finally, an “information power” approach was used to guide the sample size for the qualitative interview data collection, whereby “the more information the sample holds, relevant for the actual study, the lower number of participants is needed”. [82] (p. 1759). Given the specificity of the study aims and the purposeful sampling of participants with highly relevant knowledge and experience, it is argued that the sample size achieved sufficient information power. However, the purposeful selection of municipalities with less healthy food environments for the qualitative study may limit the generalizability of our findings. 

Nevertheless, this study makes an important contribution to the evidence base using mixed methods approaches and on the intra-urban associations between the food environment and health outcomes. A strength of this study was the rigorous approach to food outlet data validation through comprehensive cross-checking against company websites, White and Yellow page listings, and by using Google Street View. Furthermore, there was contemporaneous collection of food outlet and participant data and inclusion of both supermarket and fast food access in models to account for spatial co-occurrence. Future research at the postcode level would provide finer-grain insights, and including the “lived experience” of residents in the qualitative strand would provide additional perspectives. For example, a recent study from Spain incorporated residents’ perspectives into policy recommendations for obesity prevention [83]. 

## 5. Conclusions

This study used a mixed methods approach combining quantitative, spatial, and qualitative data to shed light on the understudied associations between local food environments and health outcomes across areas of varying suburban development in an Australian context. It was found that food outlet access was greater in established areas compared with urban-growth areas, and that fast food access was positively associated with BMI in established areas and negatively associated in growth areas. Urban design and planning policy that supports higher residential densities may influence food environments by increasing healthy food outlet access, leading to better health outcomes and reducing inequities. The methodology and findings may have utility for supporting and guiding urban planning and design policy in other rapidly growing cities worldwide.

## Figures and Tables

**Table 1:** Descriptive statistics of the preventive health survey (PHS) respondents by location: established versus urban-growth local government area.

		Location		
	Total	Established Area (*n* = 1648)	Growth Area (*n* = 1493)	*p-*Value *
Demographic Characteristics				
Age (years)				
Mean (SE)	54.15 (0.31)	56.10 (0.43)	52.48 (0.45)	<0.001
Gender (%)				
Male	38.6	38.1	38.9	
Female	61.4	61.9	61.1	0.667
Education (%)				
Primary	4.0	4.6	3.4	
Secondary	74.6	71.7	77.1	
Tertiary	21.4	23.7	19.5	<0.001
Employment status (%)				
Employed (Include self-employed)	52.2	48.6	55.2	
Unemployed	4.1	3.9	4.3	
Not in labour force	43.7	47.5	40.5	<0.001
Household income (AUD) (%)				
$0–$49,999	45.9	49.0	43.2	
$50,000–$79,999	18.1	16.8	19.3	
$80,000–$124,999	16.6	14.1	18.7	
≥$125,000	9.9	9.9	9.9	
Refused/Don’t know	9.5	10.2	8.9	<0.001
Outcome Variable				
BMI (kg/m^2^)				
Mean (SE)	27.34 (0.10)	27.10 (0.14)	27.55 (0.15)	0.030
Covariates: Behavioural				
Vegetable consumption (serves/day)				
Mean (SE)	2.26 (0.03)	2.28 (0.05)	2.24 (0.05)	0.439
Fruit Consumption (serves/day)				
Mean (SE)	1.74 (0.02)	1.79 (0.04)	1.70 (0.04)	0.032
Fast food consumption (frequency/fortnight)				
Mean (SE)	1.12 (0.04)	1.04(0.06)	1.20 (0.06)	0.053
Soft drink consumption (frequency/fortnight)				
Mean (SE)	6.12 (0.20)	5.85 (0.30)	6.35 (0.27)	0.209
Physical activity (%)				
Inactive	6.8	7.0	6.7	
Insufficient activity (Frequency & duration)	25.0	24.0	25.7	
Sufficient activity (frequency & duration)	68.2	69.0	67.6	0.602
Smoking Status (%)				
Current smoker	14.7	13.6	15.5	
Not a current smoker	85.3	86.4	84.5	0.137
Covariate: Area Level				
Area level disadvantage (IRSD)				
High disadvantage (IRSD deciles 1–3)	36.2	40.6	32.4	
Mid disadvantage (IRSD deciles 4–6)	34.6	27.9	40.3	
Low disadvantage (IRSD deciles 7–10)	29.2	31.5	27.3	<0.001

* *p*-values determined by adjusted Wald test for continuous variables and by *X*^2^ test for categorical variables. IRSD: Index of Relative Socio-Economic Disadvantage; BMI: body mass index; AUD: Australian dollar; SE: standard error.

**Table 2:** Geographic measures of supermarket and fast food chain access by location: established versus urban-growth local government area.

			Location				
	Total		Established Area (*n* = 1648)		Growth Area (*n* = 1493)		*p*-Value *
	Mean	SD	Mean	SD	Mean	SD	
Supermarket Density (Pedestrian Road Network)							
≤800 m	0.30	0.67	0.32	0.69	0.27	0.64	0.022
≤1000 m	0.49	0.86	0.52	0.86	0.47	0.86	0.128
≤1600 m	1.39	1.44	1.49	1.43	1.27	1.44	<0.001
Supermarket Density (Car Road Network)							
≤2000 m	2.11	1.79	2.30	1.84	1.89	1.72	<0.001
≤3000 m	4.57	2.91	5.13	3.20	3.96	2.42	<0.001
Fast Food Chain Density (Pedestrian Road Network)							
≤800 m	0.24	0.68	0.29	0.74	0.19	0.60	<0.001
≤1000 m	0.43	0.94	0.50	1.01	0.35	0.86	<0.001
≤1600 m	1.29	1.67	1.45	1.68	1.12	1.63	<0.001
Fast Food Chain Density (Car Road Network)							
≤2000 m	2.00	2.03	2.24	2.06	1.74	1.97	<0.001
≤3000 m	4.49	2.82	5.00	2.81	3.93	2.73	<0.001
	Median	IQR	Median	IQR	Median	IQR	
Distance to Closest Supermarket (Pedestrian Road Network) (km)	1.30	0.88–1.80	1.24	0.85–1.69	1.37	0.91–1.92	<0.001
Distance to Closest Fast Food Chain (Pedestrian Road Network) (km)	1.55	1.03–2.19	1.40	0.96–1.98	1.69	1.16–2.46	<0.001

* *p-*values determined by Analysis of Variance (ANOVA) for food outlet density and Kruskal–Wallis test for food outlet proximity. IQR: interquartile range; SD: standard deviation.

**Table 3:** Generalized estimating equations (GEE) models of associations between BMI and measures of fast food access for urban PHS respondents (*n* = 2712) by location.

					Location							
BMI (kg/m^2^) *	Total				Established Area (*n =* 1406)				Growth Area (*n* = 1306)			
	*β*	95% CI		*p-*Value	*β*	95% CI		*p-*Value	*β*	95% CI		*p*-Value
Fast Food Chain Density												
≤800 m (Pedestrian road network)	−0.019	−0.292	0.255	0.894	0.198	0.002	0.395	0.047	−0.367	−0.722	−0.012	0.043
≤1000 m (Pedestrian road network)	0.095	−0.097	0.287	0.332	0.270	0.137	0.404	0.000	−0.158	−0.443	0.127	0.278
≤1600 m (Pedestrian road network)	−0.147	−0.270	−0.024	0.019	−0.045	−0.189	0.098	0.533	−0.262	−0.431	−0.093	0.002
≤2000 m (Car road network)	-0.049	−0.125	0.027	0.205	−0.039	−0.163	0.084	0.532	-0.032	−0.150	0.086	0.593
≤3000 m (Car road network)	−0.082	−0.163	−0.001	0.046	−0.056	−0.156	0.045	0.279	−0.097	−0.258	0.065	0.241
Fast Food Chain Proximity												
(Pedestrian Road Network)	−0.016	−0.115	0.084	0.759	−0.112	−0.344	0.121	0.346	−0.053	−0.152	0.046	0.297

*Adjusted for age, gender, education level, income, vegetable intake, fruit intake, soft drink consumption, fast food consumption, physical activity, smoking, supermarket access, IRSD, and clustering at the municipality level. CI: confidence interval.

## Supplementary Materials

The following are available online at http://www.mdpi.com/1660-4601/15/7/1392/s1, Table S1: Interview guide, Table S2: GEE models of associations between BMI and measures of supermarket access for urban PHS participants by established versus urban-growth area location.
