# Supplementary material for: Local Food Environments, Suburban Development, and BMI: A Mixed Methods Study
Source: Int J Environ Res Public Health. 2018 Jul 2;15(7):1392. doi: 10.3390/ijerph15071392 (PMC6068516; doi:10.3390/ijerph15071392)
Supplement: Supplementary file 1 [file ijerph-15-01392-s001.zip › Table S2.pdf]

Table S2: GEE models of associations between BMI and measures of supermarket access for urban PHS participants by established vs urban-growth area location

BMI (kg/m^2) *	Total				Location							
					Established LGA (n = 1406)				Growth area LGA (n = 1306)			
	β	95% CI		*P* value	β	95% CI		*P* value	β	95% CI		*P* value
Supermarket density												
<= 800m (pedestrian road network)	-0.049	-0.379	0.282	0.773	0.019	-0.424	0.461	0.933	-0.162	-0.708	0.385	0.562
<= 1000m (pedestrian road network)	-0.027	-0.252	0.197	0.811	-0.006	-0.198	0.184	0.944	-0.034	-0.459	0.391	0.875
<= 1600m (pedestrian road network)	0.066	-0.118	0.251	0.480	0.040	-0.212	0.293	0.753	0.106	-0.151	0.364	0.419
<= 2000m (car road network)	-0.097	-0.242	0.049	0.194	-0.021	-0.207	0.164	0.822	-0.201	-0.427	0.024	0.080
<= 3000m (car road network)	0.009	-0.067	0.085	0.815	0.012	-0.052	0.075	0.719	0.017	-0.178	0.211	0.866
Supermarket proximity												
(pedestrian road network) (km)	-0.034	-0.391	0.323	0.851	0.025	-0.260	0.310	0.863	-0.042	-0.705	0.620	0.900

IRSD, Index of Relative Socio-economic Disadvantage; BMI= body mass index

*Adjusted for age, gender, education level, income, vegetable intake, fruit intake, frequency of soft drink consumption, frequency of fast food consumption, physical activity, smoking, fast food chain access, IRSD and clustering at the LGA level
