# Supplementary material for: Local Food Environments, Suburban Development, and BMI: A Mixed Methods Study
Source: Int J Environ Res Public Health. 2018 Jul 2;15(7):1392. doi: 10.3390/ijerph15071392 (PMC6068516; doi:10.3390/ijerph15071392)
Supplement: Supplementary file 1 [file ijerph-15-01392-s001.zip › Table S1.pdf]

Table S1: Interview Guide

**Introduction**	*In this study, local food environments are defined as are the places in an urban area or neighbourhood where people shop for food within a 20-minute walking or short driving distance from home.*
**Questions**	1. Who, if anyone, is responsible for ensuring that the community that you serve has access to a healthy local food environment?<br>2. What is the role of your organisation, if any, in influencing food environments?<br>3. In Victoria, there is an urban planning guideline for growth areas that specifies that 80-90% of households should be located within one kilometre of a town centre large enough to allow for provision of a supermarket.<br>4. Were you aware of this or any other guidelines or policies that influence the types of food environments delivered in local communities? In what way could your organisation act on these guidelines/policies? Has your organisation implemented any strategies that would address these guidelines/policies? If so, what are they?<br>5. What are the barriers, if any, to creating healthy food environments in your local community? Have you, or your organisation experienced these barriers?<br>**6.** What factors, if any, would facilitate your organisation helping to create healthy food environments in your local community?
**Quantitative & spatial data**	*Refer to 2-page report and metro Melbourne and/or local government maps from the quantitative strand.*
**Questions**	7. Based on these findings, which organisation, if any, would be responsible for acting on these findings; and what might facilitate this, if anything?<br>8. How important is having local data about food environments? What local data is most useful? How does it influence local decision-making, if at all?<br>9. What are the other influences, if any, on local decision-making about food environments?<br>10. Which other organisations - if any – might have a role in creating healthy food environments? What is their role?<br>11. Does your organisation work with this organisation? Why/ why not?<br>12. What different types of organisations need to work together to support healthier food environments? Under what circumstances might they come together to do this work? What would the co-benefits be?<br>13. What do you think are the key factors that must be in place in order to create healthy food environments?
